# Orp8 Deficiency in Bone Marrow-Derived Cells Reduces Atherosclerotic Lesion Progression in LDL Receptor Knockout Mice

**DOI:** 10.1371/journal.pone.0109024

**Published:** 2014-10-27

**Authors:** Erik van Kampen, Olivier Beaslas, Reeni B. Hildebrand, Bart Lammers, Theo J. C. Van Berkel, Vesa M. Olkkonen, Miranda Van Eck

**Affiliations:** 1 Div. of Biopharmaceutics, Cluster of BioTherapeutics, Leiden Academic Centre for Drug Research, Leiden, The Netherlands; 2 Dept. of Lipid Signalling and Homeostasis, Minerva Foundation Institute for Medical Research, Helsinki, Finland; Harvard Medical School, United States of America

## Abstract

**Introduction:**

Oxysterol binding protein Related Proteins (ORPs) mediate intracellular lipid transport and homeostatic regulation. ORP8 downregulates ABCA1 expression in macrophages and cellular cholesterol efflux to apolipoprotein A-I. In line, ORP8 knockout mice display increased amounts of HDL cholesterol in blood. However, the role of macrophage ORP8 in atherosclerotic lesion development is unknown.

**Methods and Results:**

LDL receptor knockout (KO) mice were transplanted with bone marrow (BM) from ORP8 KO mice and C57Bl/6 wild type mice. Subsequently, the animals were challenged with a high fat/high cholesterol Western-type diet to induce atherosclerosis. After 9 weeks of Western-Type diet feeding, serum levels of VLDL cholesterol were increased by 50% in ORP8 KO BM recipients compared to the wild-type recipients. However, no differences were observed in HDL cholesterol. Despite the increase in VLDL cholesterol, lesions in mice transplanted with ORP8 KO bone marrow were 20% smaller compared to WT transplanted controls. In addition, ORP8 KO transplanted mice displayed a modest increase in the percentage of macrophages in the lesion as compared to the wild-type transplanted group. ORP8 deficient macrophages displayed decreased production of pro-inflammatory factors IL-6 and TNFα, decreased expression of differentiation markers and showed a reduced capacity to form foam cells in the peritoneal cavity.

**Conclusions:**

Deletion of ORP8 in bone marrow-derived cells, including macrophages, reduces lesion progression after 9 weeks of WTD challenge, despite increased amounts of circulating pro-atherogenic VLDL. Reduced macrophage foam cell formation and lower macrophage inflammatory potential are plausible mechanisms contributing to the observed reduction in atherosclerosis.

## Introduction

ORPs are Oxysterol binding protein Related Proteins and comprise an evolutionary conserved family of 12 members. Although the family as a whole has a range of functions, a unifying theme is that ORPs can act as sterol sensors communicating with different signaling pathways [1]. Sterol signaling mediates several processes important to macrophage function in atherosclerosis, such as macrophage cholesterol efflux, pro-inflammatory signaling and the ability of macrophages to differentiate to functionally diverse subtypes [2], [3], [4]. Previous studies have demonstrated that altering the expression of ORPs in macrophages results in changes in cellular function and atherosclerosis susceptibility. Silencing of ORP1L reduced cholesterol efflux from macrophages, while overexpression of ORP1L led to increased atherosclerosis development in Low-Density Lipoprotein receptor knockout (LDLr KO) mice [5], [6]. ORP3 overexpression inhibits phagocytosis by macrophages, and macrophage intracellular cholesterol transport is disrupted in cells deficient in ORP5 [7], [8]. Furthermore, mutations in the ORP7 gene are associated with increased LDL and total cholesterol, risk factors for atherosclerosis [9]. Additionally, ORP8 is a potentially interesting target for atherosclerosis research.

In humans, mutations in the ORP8 gene are associated with changes in High-Density Lipoprotein (HDL) cholesterol levels in blood [10]. Furthermore, ORP8 is expressed by macrophages in human atherosclerotic lesions [11]. THP-1 macrophages in which ORP8 has been knocked down display an increased ability to efflux cholesterol to nascent HDL particles due to increased expression of ATP-Binding Cassette transporter A1 (ABCA1), which is essential for cholesterol transport to lipid-poor apoAI, the primary apolipoprotein of HDL [11]. Similarly, female ORP8 KO mice display a markedly increased expression of ABCA1 in the liver, as well as increased levels of HDL cholesterol and ApoAI particles in blood [12], [13]. This indicates a role for ORP8 as a regulator of cellular cholesterol metabolism and efflux.

The aim of the current study is to investigate the effects of ORP8 deletion in macrophages on atherosclerotic lesion development. Hereto irradiated LDLr KO mice were transplanted with ORP8 KO bone marrow (BM) and fed a high fat/high cholesterol Western-Type Diet (WTD) for periods of 6 and 9 weeks. We show that mice receiving ORP8 KO BM developed smaller atherosclerotic plaques despite increased levels of pro-atherogenic lipoproteins and propose a possible mechanism for the observed decrease in atherosclerosis.

## Materials and Methods

### Perivascular Collar Placement

LDL receptor knockout (LDLr KO) mice were obtained from the Jackson Laboratory and bred at the Gorlaeus Laboratories in Leiden, the Netherlands.

To assess gene expression during atherosclerotic plaque development, mice were fed a high-fat/high-cholesterol Western-Type Diet (WTD), containing 15% cacao butter and 0.25% cholesterol (WTD; Special Diet Services) for 2, 4, 6, 8, 10 and 12 weeks. At two weeks a baseline group was sacrificed and atherosclerotic carotid artery lesions were induced by perivascular collar placement as described previously [14]. At the end of the WTD feeding period the mice were anaesthetized using a mix of rompun, ketamine and atropine at a lethal dose. Mice were then exsanguinated and perfused with PBS.

Animal experiments were approved by the Ethics Committee for Animal Experiments of Leiden University (permit number 08015) and performed at the Gorlaeus Laboratories of the Leiden Academic Center for Drug Research in accordance with the National Laws and the Directive 2010/63/EU of the European Parliament.

### Bone Marrow-Derived Macrophage Generation and Culturing

Bone Marrow was isolated from ORP8 KO male mice (age 10 weeks). Bone marrow cells were plated in DMEM/20% FCS/1% penicillin/1% streptomycin and differentiated into macrophages by addition of 30% L929 cell-conditioned media (as a source of M-CSF) for 7 days according to Van Eck et al. [15] Macrophages were incubated for 24 h in cell culture plates (Greiner Bio-One) the absence or presence of 100 ng/mL LPS (Salmonella Typhosa, Sigma) or 20 ng/mL IL-4 (Sigma) and subsequently lysed for mRNA extraction or analyzed by FACS.

### Flow Cytometry Analysis

BMDMs were stained using antibodies against CD40 and CD80 (eBioscience) and analyzed on a FACS Canto II (BD Biosciences, Mountain View, CA).

### Cytokine Quantification

Concentrations of murine TNFα and IL-6 in the supernatant of BMDMs were assayed using an ELISA kit according to the manufacturer's protocol (BD Bioscience). Absorbance was detected at 450 nm.

### mRNA Expression Analysis by Microarray and Real Time PCR

Carotid arteries with collar-induced plaques of female LDLr KO mice which were fed WTD for 2, 4, 6, 8, 10 and 12 weeks were isolated. Samples were digested with proteinase K and mechanically homogenized. Total RNA from samples was isolated using the guanidinium thiocyanate (GTC) method [16] and a microarray was performed by Service XS (Leiden, The Netherlands).

Furthermore, total RNA from Bone Marrow-Derived Macrophages (BMDMs) was isolated using the same GTC method and reverse transcribed using a RevertAid M-MuLV enzyme (Fermentas, Burlington, Canada). The mRNA expression levels were assessed by real time PCR (ABI PRISM 7500; Applied Biosystems, Foster City, CA) using SYBR Green technology (Applied Biosystems). The average of GAPDH and 36B4 or ß-actin expression was used as a housekeeping control.

### Bone Marrow Transplantation

To study the effect of ORP8 deletion in hematopoietic cells, bone marrow (BM) of male ORP8 KO mice and WT controls (age 12 weeks) was obtained from the laboratory of Dr. Olkkonen (Minerva Foundation Institute for Medical Research, Helsinki, Finland) for transplantation to female LDLr KO mice (age 12 weeks) at the Leiden Academic Centre for Drug Research. In short, 5×10^6^ bone marrow cells were injected into the tail vein of lethally irradiated recipients [15].

After 8 weeks recovery, recipient mice were fed WTD for 6 and for 9 weeks. At the end of the WTD feeding period the mice were anaesthetized using a mix of rompun, ketamine and atropine at a lethal dose, after which peritoneal cells were isolated by peritoneal lavage with PBS. Mice were then exsanguinated, perfused with PBS, and organs were harvested.

### Serum cholesterol, triglycerides and cytokines

Serum concentrations of free cholesterol were determined by enzymatic colorimetric assays with 0.048 U/mL cholesterol oxidase (228250, Calbiochem) and 0.065 U/mL peroxidase (P8375, Sigma) in reaction buffer (1.0 KPi buffer, pH = 7.7 containing 0.01 M phenol, 1 mM 4-amino-antipyrine, 1% polyoxyethylene-9-laurylether, and 7.5% methanol). For the determination of total cholesterol, 0.03 U/mL cholesteryl esterase (228180, Calbiochem) was added to the reaction solution. Absorbance was read at 490 nm. Serum triglycerides were measured using a colorimetric assay (Roche Diagnostics, Mannheim Germany) according to the manufacturer's instructions. The distribution of cholesterol and triglycerides over the different lipoproteins in serum was determined by fractionation of 25 µL of serum of each mouse using a Superose 6 column (3.2×300 mm, Smart-System; Pharmacia, Uppsala, Sweden). Total cholesterol content of the effluent was determined as described above. Concentrations of murine TNFα and IL-6 in the plasma were assayed using an ELISA kit according to the manufacturer's protocol (BD Bioscience). Absorbance was detected at 450 nm.

### Blood Cell Analysis

An automated Sysmex XT-2000iV Veterinary Haematology analyzer (Sysmex Corporation) was used to analyze leukocyte numbers and composition in blood and peritoneal leukocyte samples.

### Macrophage cholesterol efflux studies

Bone marrow-derived macrophages were incubated with 0.5 µCi/mL ^3^H-cholesterol in DMEM/0.2% free fatty acid free BSA for 24 hours at 37°C. To determine cholesterol loading, cells were washed three times with washing buffer (50 mmol/L Tris, containing 0.9% NaCl, 1 mmol/L EDTA, 5 mmol/L CaCl_2_, pH 7.4), lysed in 0.1 mol/L NaOH, and the radioactivity was determined by liquid scintillation counting. Cholesterol efflux was studied by incubating the cells with DMEM/0.2% BSA alone, or with DMEM/0.2% BSA supplemented with either 10 µg/mL apolipoprotein AI (Calbiochem) or 50 µg/mL human HDL (isolated according to Redgrave, as described previously [17]). Radioactivity in the medium was determined by scintillation counting after 24 hours of incubation. Cholesterol efflux was defined as (dpm_medium_/dpm_cell_+dpm_medium_)×100%.

### Histological Analysis of the Aortic Root

Serial sections (7 µm) of the aortic root were cut using a Leica CM3050S cryostat and stained for lipids using oil red-O. The atherosclerotic lesion areas in oil red-O stained cryostat sections of the aortic root were quantified using the Leica image analysis system, consisting of a Leica DMRE microscope coupled to a video camera and Leica Qwin Imaging software (Leica Ltd, Cambridge, UK). Mean lesion area (in µm^2^) was calculated from 10 consecutive oil red-O stained sections, starting at the appearance of the tricuspid valves. For the assessment of the macrophage area, sections were immunolabeled with MOMA-2 (Research Diagnostics Inc; dilution 1∶50). The MOMA-2-positive lesion area was subsequently quantified using the Leica image analysis system and expressed as a fraction of total lesion area. Apoptosis in the plaque was visualized using an *In-Situ* Cell Death Detection Kit (POD) (11 684 817 910, Roche) as per manufacturer's instructions. The tissue was counter stained using 0.3% Methyl Green for 2 minutes. Methyl Green positive nuclei and TUNEL positive nuclei in the plaque were counted and the fraction of TUNEL positive nuclei of total Methyl Green positive nuclei was calculated. Furthermore, presence of CD3^+^ T cells in aortic root sections was visualized using an anti-CD3 antibody (RM-9107, Thermo Scientific; dilution 1∶150). Leica Qwin software was used to quantify adventitia size in the stained sections. CD3^+^ T cells were quantified and normalized for adventitia size. A Masson Trichrome kit consisting of Biebrich Scarlet Acid Fuchsin, Phosphotungstic Acid, Phosphomolybdic Acid and Aniline Blue was used according to manufacturer's instructions (Procedure HT15) to visualize plaque collagen.

### Statistical Analysis

Statistically significant differences were tested with the appropriate tests as indicated using Graphpad Prism software. The probability level (alpha) for statistical significance was set at 0.05. Results are expressed as an average ± SEM.

## Results

### Expression of ORP8 is detectable in the atherosclerotic plaque and correlates with expression of macrophage marker CD68

**Figure 1:**
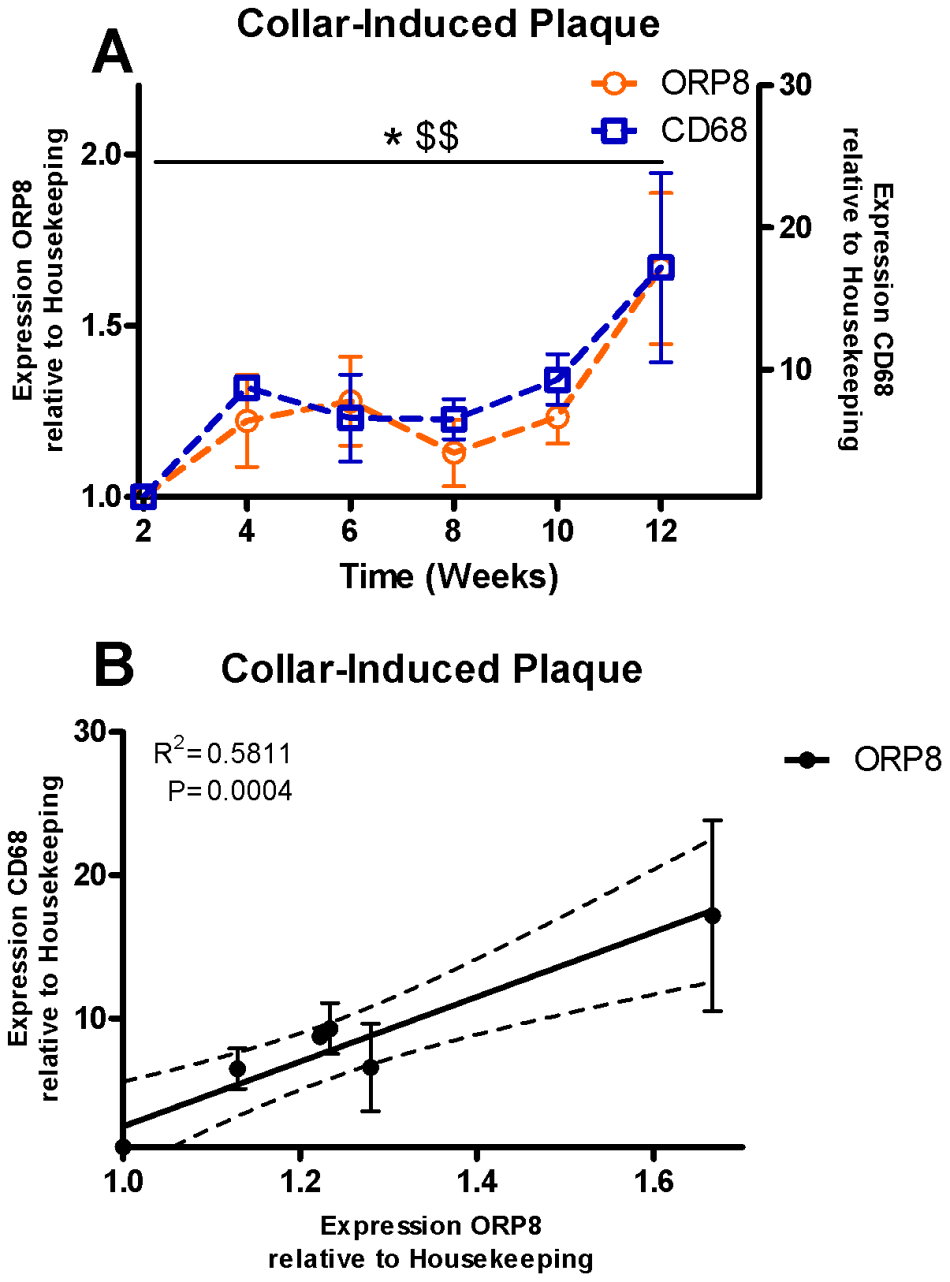
ORP8 expression detected in murine atherosclerotic plaques correlates with expression of CD68. mRNA was isolated from collar-induced plaques located in the carotid artery of LDLr KO mice. Samples were taken at 2 week intervals from week 2 baseline until week 12. Expression of ORP8 and the monocyte/macrophage marker CD68 was measured at each timepoint by microarray analysis and normalized for expression at baseline. A) ORP8 was found to be upregulated during plaque progression, as was CD68 (* P<0.05; $$<0.01, respectively). N = 3, values are expressed as mean ± SEM. Significance was assessed by ANOVA with Dunnetts post-test. B) Expression of ORP8 in murine atherosclerotic plaques correlated with expression of CD68 (P<0.001). Significance was assessed by linear regression.

Atherosclerotic plaques were induced in the carotid artery of LDLr KO mice by surgically placing a collar around the carotid artery and subjecting the animals to an atherogenic Western-Type Diet (WTD) for up to 12 weeks. Animals were sacrificed every two weeks starting at two weeks after collar placement. RNA was isolated from the carotid arteries with atherosclerotic lesions and analyzed by microarray. RNA expression of ORP8 was increased during atherosclerosis progression up to 66% after 12 weeks of WTD feeding (P<0.05, figure 1A). Expression of CD68, indicative of macrophage infiltration, in the collar-induced plaques followed a strikingly similar pattern to the expression pattern of ORP8. At 12 weeks of WTD feeding expression of CD68 was increased 17-fold (P<0.01, figure 1A). Relative expression of ORP8 and CD68 were plotted against each other, and linear regression analysis showed a correlation between ORP8 and CD68 expression in the atherosclerotic carotid arteries, indicating that ORP8 is primarily expressed by macrophages in the murine lesions (P<0.001, figure 1B).

### Decreased expression of macrophage differentiation markers, pro-inflammatory cytokines and co-stimulatory molecules by ORP8 KO BMDMs

**Figure 2:**
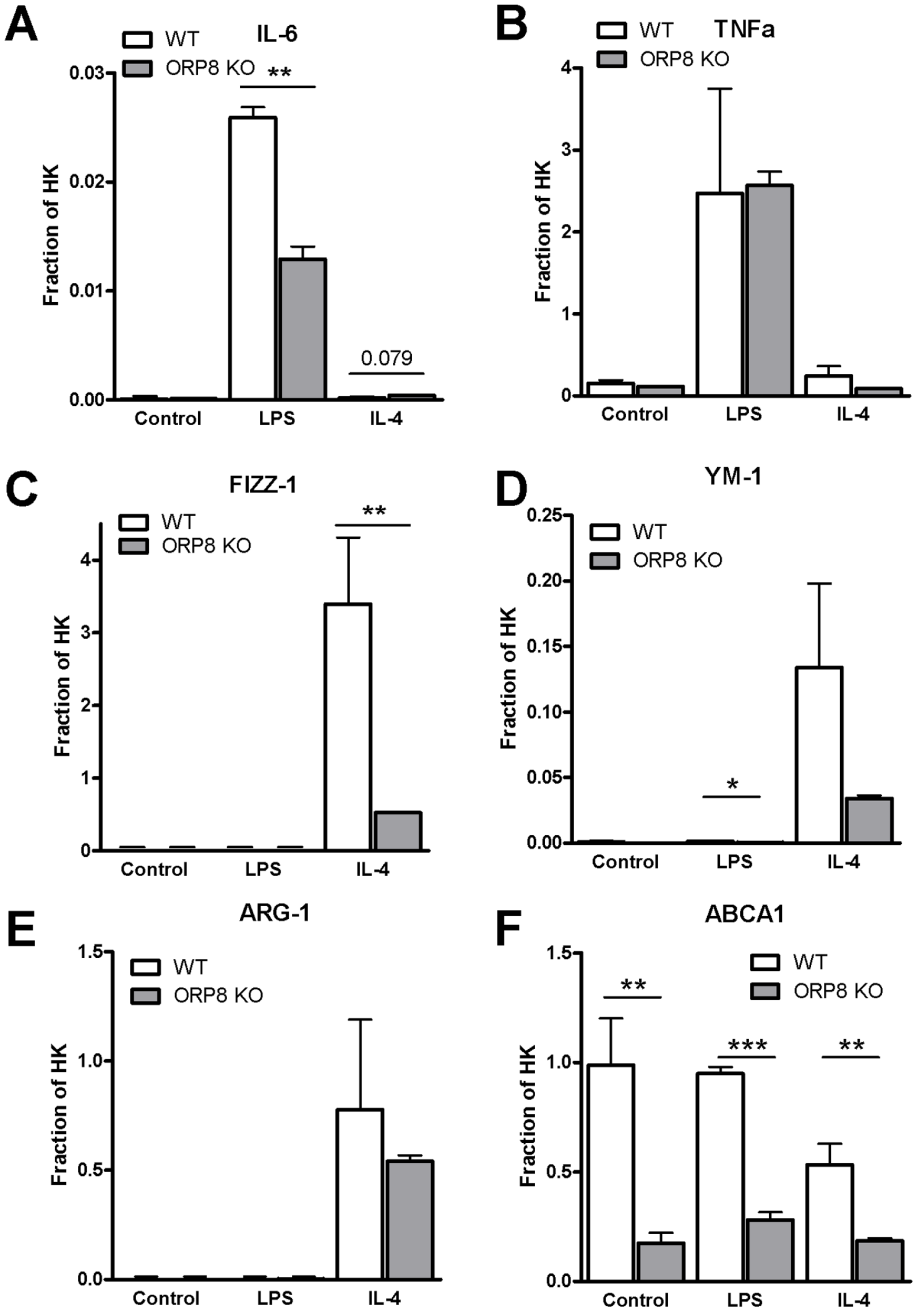
ORP8 KO BMDMs display reduced expression of macrophage differentiation factors and excrete less of pro-inflammatory cytokines. Bone Marrow-Derived Macrophages (BMDM) were generated from bone marrow of ORP8 KO mice or WT controls and incubated with LPS (100 ng/mL), IL-4 (20 ng/ml) or PBS. Expression of macrophage differentiation markers was measured by QPCR and calculated as relative to housekeeping (HK) for the M1 markers IL-6 (A) and TNFα (B), the M2 markers FIZZ-1 (C), YM-1 (D), Arg1 (E), and ABCA1 (F) (N = 3). Significance was determined by Student T-test, data are expressed as mean ±SEM. Statistical significant difference * P<0.05, ** P<0.01, *** P<0.001 as compared to controls.

Next, the effects of ORP8 deficiency on macrophage gene expression were investigated. Macrophages are capable of differentiating into a range of subtypes which vary widely in inflammatory potential and wound healing ability, the extreme ends of the subtype distribution being the pro-inflammatory M1 and the anti-inflammatory M2 macrophages [18], [19], [20]. BMDMs were cultured from bone marrow of male ORP8 KO mice and stimulated with LPS, IL-4 or left unstimulated to generate M1, M2 or naïve macrophages, respectively. At 24 hours after stimulation RNA was isolated and expression of macrophage differentiation markers was measured. As anticipated, stimulation with LPS induced the expression of the M1 markers IL-6 and TNFα, while IL-4 stimulation led to augmented expression of the M2 markers FIZZ-1, YM-1, and Arginase 1 (Arg1) (figure 2A–E). In LPS-stimulated ORP8 KO BMDMs, a significant 2-fold decrease in expression of the M1 marker IL-6 was found (P<0.01, figure 2A), while no differences in expression of TNFα could be found (figure 2B). Expression of the M2 marker FIZZ-1 was decreased 6-fold in IL-4 stimulated ORP8 KO BMDMs (P<0.01, figure 2C). There was also a trend towards a decrease in expression of YM-1 in ORP8 KO BMDMs in all treatment groups. However, this only reached significance in the LPS-stimulated cells (P<0.05, figure 2D), but expression of YM-1 under these conditions is extremely low. No significant difference was found in the expression of Arg1 (figure 2E). Expression of ABCA1 was measured, as ORP8 has previously been shown to regulate expression of ABCA1 [11]. There was a strong decrease in expression of ABCA1 in naïve macrophages and macrophages stimulated with LPS and IL-4 (5-fold P<0.01, 3.4-fold P<0.001 and 2.9-fold P<0.01, respectively, figure 2F).

**Figure 3:**
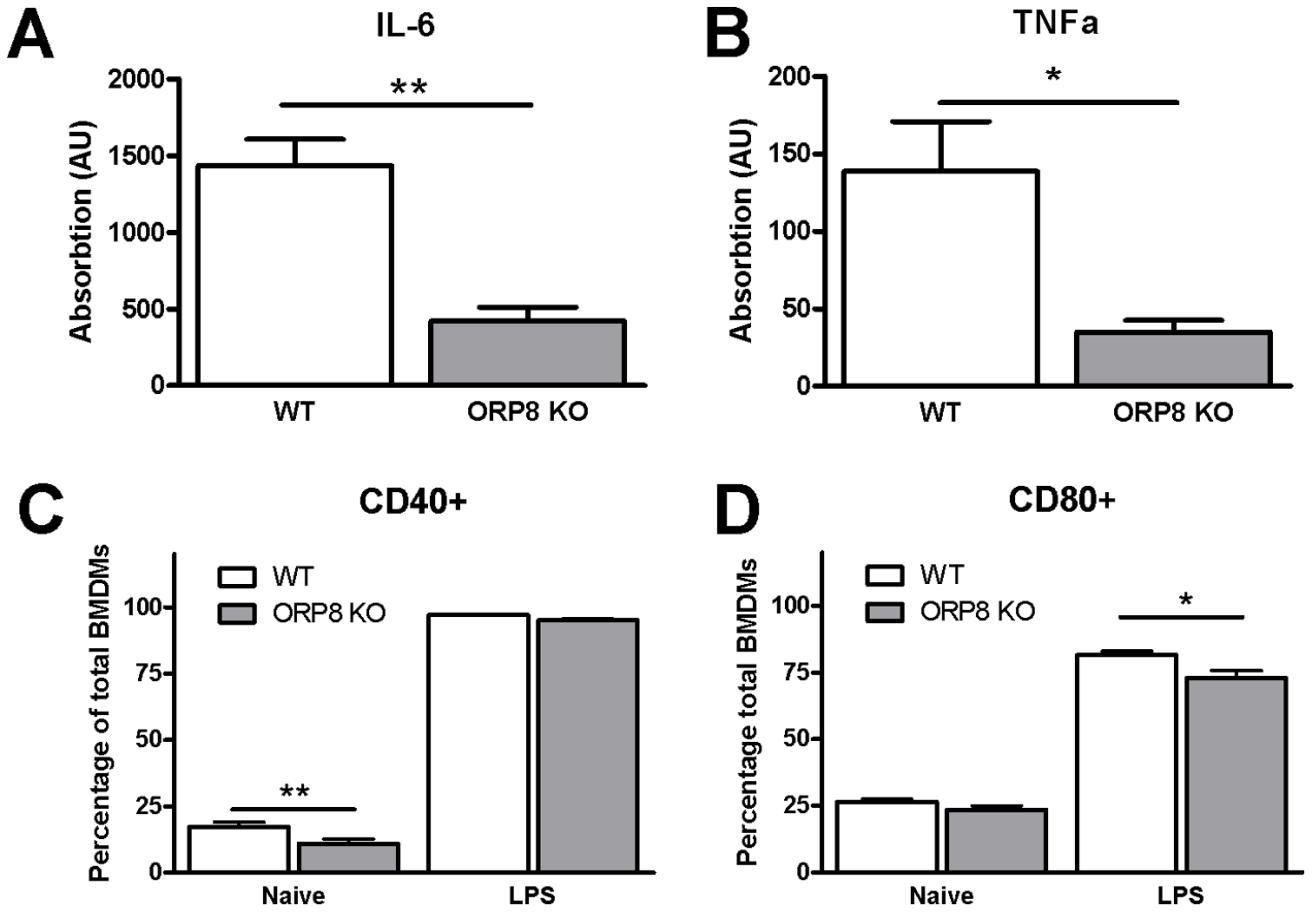
ORP8 KO BMDMs have reduced excretion of pro-inflammatory cytokines and reduced expression of co-stimulatory factors. Bone Marrow-Derived Macrophages (BMDMs) were generated from bone marrow of ORP8 KO mice or WT controls and stimulated with LPS (100 ng/mL). A) A decrease in secretion of IL-6 by LPS stimulated ORP8 KO BMDMs was measured using ELISA. (** P<0.01, N = 4) B) Similarly, TNFα secretion was also decreased in LPS-stimulated ORP8 KO BMDMs. (* P<0.05, N = 4). Expression of co-stimulatory factors CD40 and CD80 on BMDMs from ORP8 BM and WT BM was measured using FACS C) The percentage of unstimulated BMDMs expressing CD40 was decreased in the ORP8 KO group. (** P<0.01, N = 5) D) After stimulation with LPS, the percentage of BMDMs expressing CD80 was decreased in the ORP8 KO group (* P<0.05, N = 5). Significance of ELISA results was determined by Student T-test. Significance of FACS results was assessed by two-way ANOVA with Bonferroni post-tests. Data are expressed as mean ± SEM.

In addition to mRNA expression analysis, cytokine concentrations in supernatant of LPS stimulated BMDMs were measured by ELISA, showing decreased production of the pro-inflammatory cytokines IL-6 (3.5-fold, P<0.01) and TNFα (4-fold, P<0.05) by the ORP8 KO cells (figure 3A–B). Furthermore, the percentage of unstimulated BMDMs expressing CD40 was decreased in the ORP8 deficient group (0.6-fold, P<0.01), while there was also a small, but significant decrease (0.9-fold, P<0.05) in the amount of CD80 expressing ORP8 KO BMDMs after stimulation with LPS, compared to WT BMDMs (figure 3C–D).

### Increased Cholesterol and Triglycerides in the VLDL fraction of blood from ORP8 KO BM recipient mice

**Figure 4:**
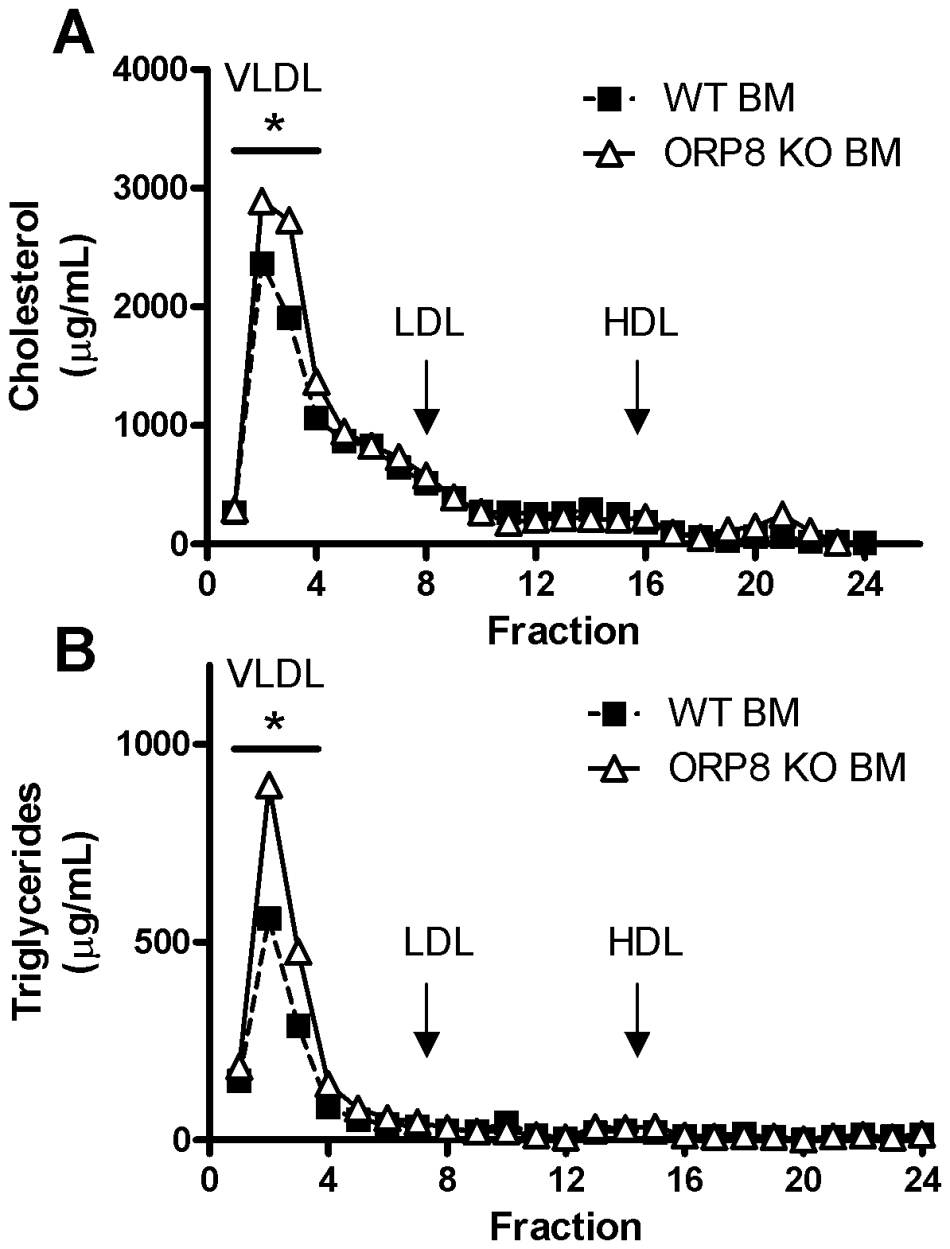
Increased VLDL cholesterol and triglycerides in LDLr KO mice receiving ORP8 KO bone marrow. Blood was isolated from LDLr KO mice after transplantation with ORP8 KO bone marrow (BM) or Wild Type (WT) BM. Lipoproteins were fractioned by size using FPLC and cholesterol and triglyceride contents were measured in each fraction. A) 17 weeks after transplantation and after 9 weeks of WTD feeding ORP8 KO BM recipients exhibit increased VLDL cholesterol (* P<0.05) B) Similarly, an increase in triglycerides in the VLDL fraction in blood of ORP8 KO BM recipients on WTD was found (* P<0.05). Significance was determined by Student t-test. N = 7, data are expressed as mean ±SEM.

To study the effect of macrophage ORP8 deletion on atherosclerosis, bone marrow from ORP8 KO mice and WT mice was transplanted into recipient LDLr KO animals. The recipients were then fed a chow diet for 8 weeks and consecutively WTD for a maximum of 9 weeks. No difference in body weight was observed between the groups throughout the experiment. At the end of both periods of dietary feeding blood was taken for lipid analysis (table 1). Although there was a small increase in free cholesterol in ORP8 KO BM recipients on chow diet at 18 weeks after transplantation, no difference between the groups was found after 6 and 9 weeks of WTD feeding. However, at 9 weeks of WTD feeding a 1.4-fold increase in plasma triglycerides was found in the ORP8 KO BM recipient mice (P<0.05, table 1). Serum from animals after 9 weeks of WTD feeding was fractionated by FPLC and analyzed for cholesterol and triglyceride content. VLDL cholesterol was increased 1.2-fold in the ORP8 KO BM recipients (P<0.05, figure 4A). While there was also a 1.2-fold increase in total cholesterol, this failed to reach statistical significance. Additionally, a 1.6-fold increase in VLDL triglycerides was found (P<0.05, figure 4B). Following the observation that IL-6 and TNFα secretion were decreased in ORP8 KO BMDMs, IL6 and TNFα were measured in blood samples of the BM recipients. However, the concentration of these cytokines was below the threshold of detection (data not shown).

**Table 1:** After 6 and 9 weeks of WTD feeding no difference in blood cholesterol levels was found between LDLr KO mice receiving ORP8 KO BM or WT BM.

	0 weeks		6 weeks		9 weeks	
	WT BM	ORP8 KO BM	WT BM	ORP8 KO BM	WT BM	ORP8 KO BM
Body Weight (gr)	25.7 (±0.4)	26.8 (±1.0)	29.3 (±1.3)	27.2 (±0.7)	27.1 (±0.9)	27.2 (±0.6)
Triglycerides (µg/ml)	-	-	-	-	1263 (±95)	1794 (±166)*
Free Cholesterol (µg/ml)	678 (±20)	745 (±19)	3966 (±259)	4151 (±203)	3665 (±194)	3749 (±229)
Esterified Cholesterol (µg/ml)	4467 (±175)	4468 (±138)	9336 (±1003)	11577 (±1552)	12901 (±759)	14185 (±1129)
Total Cholesterol (µg/ml)	5148 (±189)	5213 (±139)	13302 (±1273)	15727 (±1723)	16566 (±939)	17934 (±1351)

Body weight was measured during the chow feeding and the WTD feeding period, but no difference between the groups was found. At the end of the 9 weeks WTD feeding period ORP8 KO BM recipients had increased blood triglycerides (TG) (N = 15, *P<0.05). Furthermore, free cholesterol (FC), cholesteryl esters (CE) and total cholesterol (TC) were measured in WT BM and ORP8 KO BM recipients on chow diet and after 6 and 9 weeks on WTD. A small increase in FC was observed in the ORP8 KO BM recipients on chow diet, but no differences could be found after WTD feeding (N = 11, * P<0.05, significance was assessed by Student T-test with Welsh correction if required). Results are expressed as mean ± SEM.

### No change in circulating leukocytes, but increased amounts of foam cells in the peritoneal cavity

**Figure 5:**
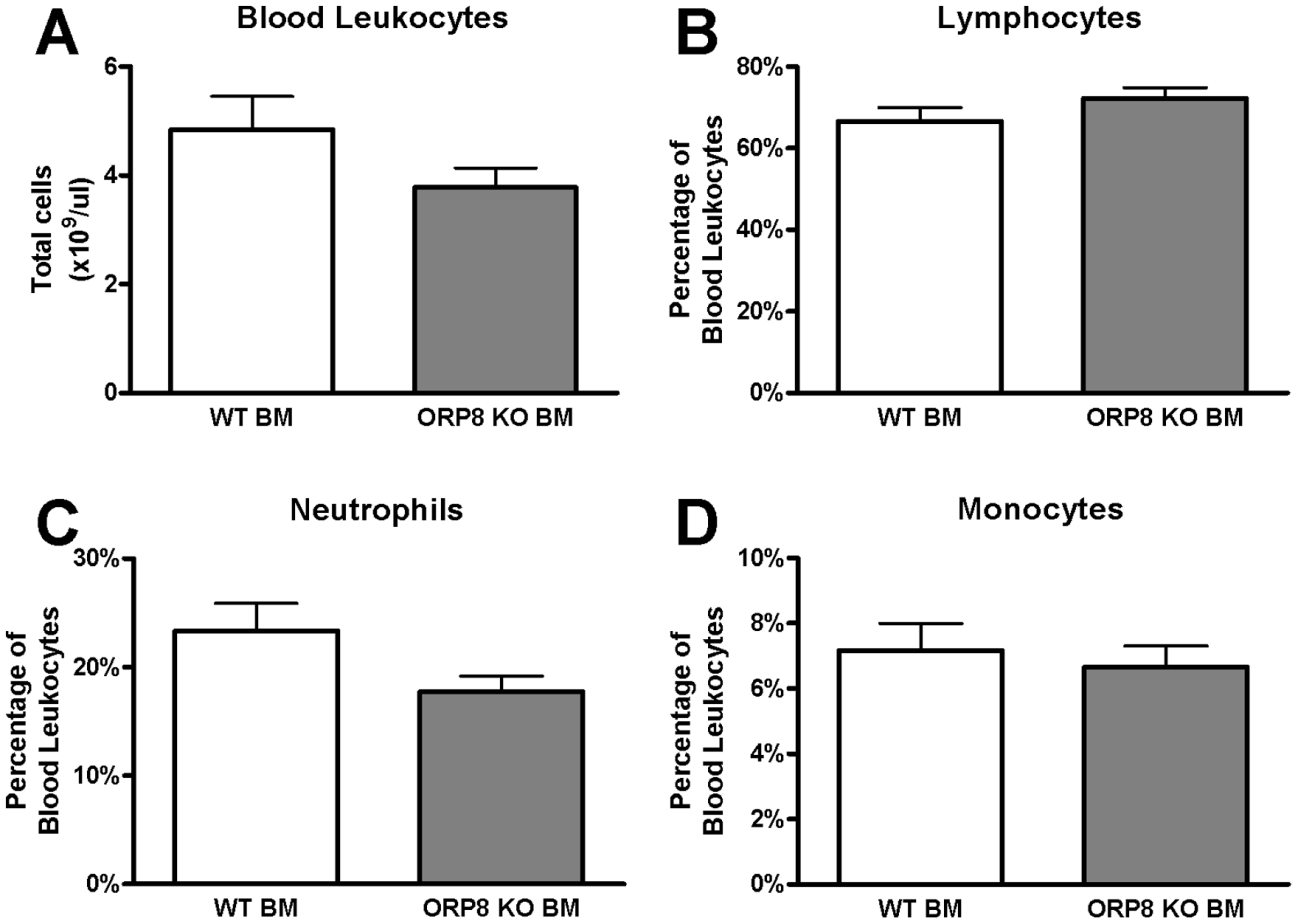
No effect on blood leukocyte counts in LDLr KO mice receiving ORP8 KO bone marrow. At 17 weeks after transplantation and after 9 weeks of WTD feeding, blood was isolated from bone marrow recipient animals. Blood leukocytes were counted using a hematology analyzer, and different subpopulations were quantified as percentage of total leukocytes. A) No difference between the ORP8 KO BM recipients and the wildtype (WT) BM recipients in the amount of circulating leukocytes. B–D) Similarly, there was no difference in the percentage of lymphocytes, neutrophils and monocytes of total leukocytes. N = 15, results are expressed as mean ±SEM and significance was assessed by Student T-test.

After 9 weeks of WTD feeding, blood samples and peritoneal lavage from LDLr KO mice transplanted with ORP8 KO BM or WT BM were analyzed for leukocyte content. The number of circulating leukocytes was not changed between the WT BM recipients and the ORP8 KO BM recipients (figure 5A). Furthermore, there was no detectable difference in leukocyte composition, as the proportion of lymphocytes, monocytes and neutrophils in blood were all unchanged (figure 5B–D).

**Figure 6:**
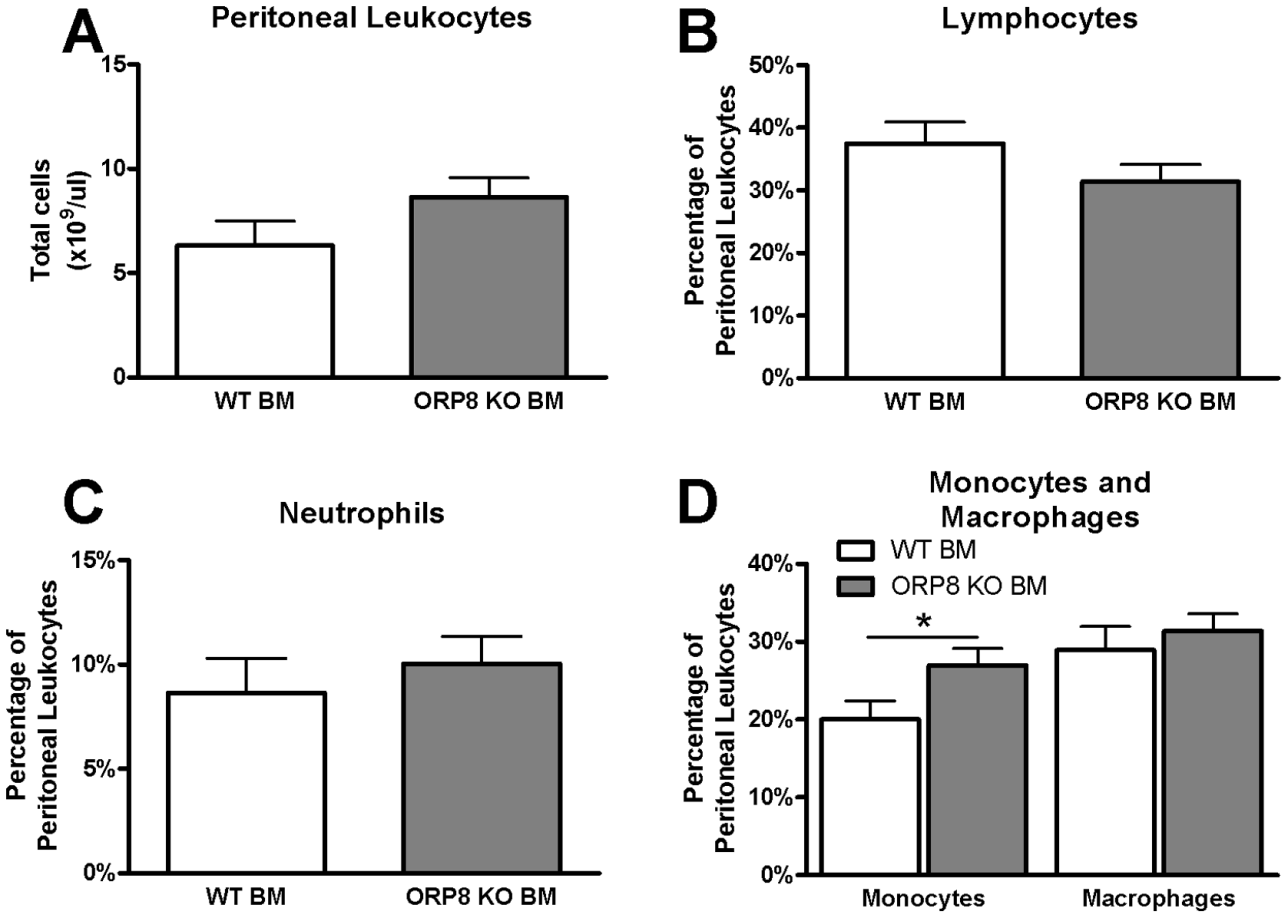
Increase in peritoneal monocytes in LDLr KO mice receiving ORP8 KO BM, but decrease in peritoneal foam cells. Peritoneal leukocytes were isolated from ORP8 KO BM recipients and wild type BM recipients and counted using a hematology analyzer at 17 weeks after BMT and 9 weeks WTD feeding. A) No difference between treatment groups was found in the total number of peritoneal leukocytes. B) The amount of peritoneal lymphocytes did not differ between the groups. C) Similarly, there was no difference between the groups in the amount of neutrophils in the peritoneum. D) However, there was an increase in the percentage of peritoneal monocytes out of total leukocytes in the ORP8 KO BM recipients, but no difference in macrophage content (* P<0.05). N = 15, significance was assessed by Student T-test. Results are expressed as mean ±SEM.

Similarly, no difference in the total amount of leukocytes and the proportion of lymphocytes and neutrophils was found in the peritoneal cavity (figure 6A–C). However, there was a significant 35% increase in the proportion of peritoneal monocytes, but not macrophages (P<0.05, figure 6D).

### Decreased foam cell formation in the presence of reduced ABCA1 expression in peritoneal cells of ORP8 KO BM recipients, but unaffected macrophage cholesterol efflux

**Figure 7:**
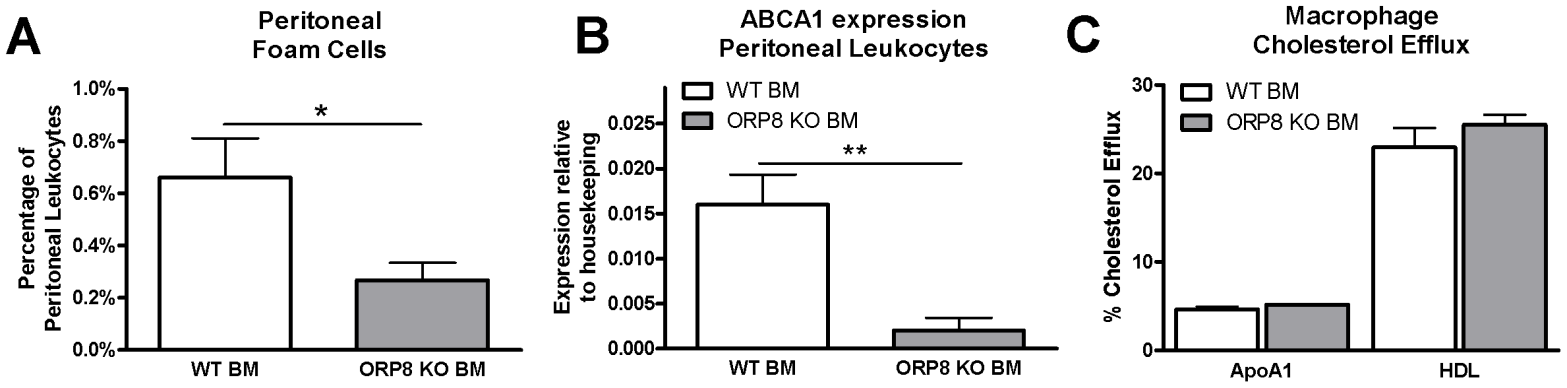
Reduced peritoneal foam cell formation and lower expression of ABCA1 in ORP8 KO macrophages, but no change in cellular cholesterol efflux. Expression of ABCA1 was measured by QPCR in Peritoneal Leukocytes isolated from LDLr KO mice at 17 weeks after transplantation with WT BM or ORP8 KO BM and 9 weeks WTD feeding. A) The ORP8 KO transplanted group displays a reduced amount of foam cells when expressed as a percentage of total peritoneal leukocytes. B) In line, expression of ABCA1 in peritoneal leukocytes of ORP8 KO BM recipients is reduced. (** P<0.01, N = 4) C) However, no change in cholesterol efflux of BMDMs to ApoA1 or HDL was found (N = 5). Results are expressed as mean ± SEM after subtraction of baseline efflux to BSA. Significance was determined by Student t-test.

Interestingly, although no difference in the amount of peritoneal macrophages was found, the amount of foam cells in the peritoneal cavity was strongly decreased (2.5-fold P<0.05, figure 7A). Additionally, in line with the reduced expression of ABCA1 in ORP8 KO BMDMs (figure 2F), ABCA1 expression in *ex-vivo* peritoneal leukocytes from ORP8 KO BM recipients was reduced 8-fold (P<0.01, figure 7B). However, no difference in cholesterol efflux from ^3^H-cholesterol labeled BMDMs to ApoA1 or to HDL was observed (figure 7C), indicating that the level of ABCA1 expression remained sufficiently high to maintain proper macrophage cholesterol efflux.

### Reduced atherosclerotic plaque size after 9 weeks Western-type diet challenge, together with increased macrophage area in plaques of ORP8 KO BM recipients

**Figure 8:**
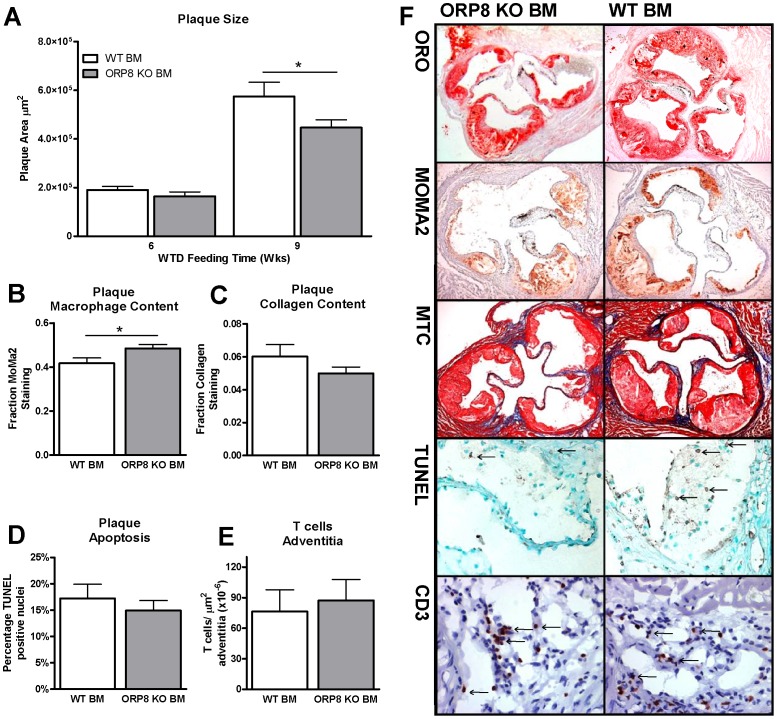
Decreased plaque size in ORP8 KO BM recipients and increased plaque macrophage content after 9 weeks WTD feeding. A) Plaque size was quantified in Oil Red O stained sections. After 9 weeks of WTD feeding following BMT, a decrease in plaque size could be measured. No difference was observed after 6 weeks of dietary challenge. (N = 13 * P<0.05, data was analyzed by two-way ANOVA with Bonferroni post-tests) B) Plaque macrophages were visualized using MoMa2 staining and quantified as a percentage of total plaque area. After 9 weeks of WTD feeding, an increase in the MoMa2 positive fraction was detected in the ORP8 KO BM recipients. (N = 13 * P<0.05, data was analyzed by two-way ANOVA with Bonferroni post-tests) C) Collagen was visualized by staining plaques from ORP8 BM and WT BM recipients with a Masson's Trichrome (MTC) kit, but no difference was found between the groups (N = 15, significance was assessed by Student T test). D) Apoptotic cells in atherosclerotic plaques of ORP8 KO BM recipients and WT BM controls were visualized by TUNEL staining. No difference in the amount of apoptotic cells was detected between treatment groups (N = 8, significance was assessed by Student T test). E) Aortic root sections from ORP8 KO BM recipients and WT BM controls were stained for CD3 to detect T cells. No difference between the groups was detected (N = 8, significance was assessed by Student T test). F) Representative images of Oil-Red O (ORO), MoMa2, TUNEL, CD3 and MTC stainings performed on atherosclerotic plaques of mice receiving ORP8 KO BM or WT BM. Images of ORO, Moma2 and MTC stainings, original magnification 5×. Images of TUNEL and CD3 stainings, original magnification 20×.

Despite the observed increased VLDL cholesterol and triglycerides in the blood of the ORP8 KO BM recipients, Oil Red-O staining of atherosclerotic plaques revealed a 20% reduction in plaque size after 9 weeks of WTD feeding (P<0.05, figure 8A, F). No significant difference in plaque size was found after 6 weeks of WTD feeding, indicating that macrophage ORP8 deficiency did not significantly affect development of early atherosclerotic plaques of less than 200.000 µm^2^. Further morphological analysis by MOMA-2 staining of atherosclerotic plaques after 9 weeks of WTD challenge revealed that a 20% larger fraction of the plaques in the ORP8 KO BM recipients was made up of macrophages (P<0.05, figure 8B, F). Sections were stained with a Masson's Trichrome kit to visualize collagen deposition in the plaques. However, no difference was found (figure 8C, F). Moreover, TUNEL staining revealed no difference in the amount of apoptotic cells in the plaque, indicating that deficiency of ORP8 in macrophages did not result in differences in cell death by apoptosis. (figure 8D, F). *In vitro* studies using BMDMs indicated decreased inflammatory potential of macrophages lacking ORP8, as evidenced by a reduced secretion of cytokines IL-6 and TNFα (figure 2G–H). Therefore also a CD3 staining was carried out to quantify the T cells in the adventitia underlying the atherosclerotic plaque, but no difference was found between the groups (figure 8E, F).

## Discussion

In the current study we showed that ORP8 expression is increased during atherosclerotic lesion development, a process which coincides with macrophage infiltration. This corresponds with the finding that ORP8 is present in human atherosclerotic plaques as opposed to healthy arteries, and is produced there by macrophages [21]. To investigate the role of macrophage ORP8 in atherosclerotic lesion development we transplanted ORP8 knockout (KO) bone marrow into atherosclerosis susceptible LDLr KO mice. Deletion of ORP8 in bone marrow-derived cells resulted in increased levels of VLDL cholesterol and triglycerides in the blood, but also a decrease in atherosclerotic plaque size.

ORP8 KO animals on a C57Bl/6 background exhibit increased levels of HDL cholesterol in the blood, and increased ApoAI production by hepatocytes [13]. Upon transplantation of ORP8 KO bone marrow into LDLr KO mice, we did not find any differences in HDL cholesterol in the blood of the BM recipients. This is not surprising, as the contribution of macrophages to HDL formation is minor [22]. It is thus likely that the HDL phenotype observed previously in the total body ORP8 KO animals is primarily hepatocyte driven. In contrast, we did observe a marked increase in VLDL cholesterol and triglycerides in ORP8 KO BM recipient mice on WTD. Interestingly, also in total body ORP8 KO mice a strong tendency towards an increase in blood triglycerides was observed, which in the female mice could be ascribed to an increase in the VLDL fraction [13]. This change was attributed to reduced lipoprotein lipase activity and possible changes in SREBP signaling [13], [23]. Hence, it is tempting to speculate that the increase in VLDL triglycerides in the total body ORP8 KO mice is the consequence of alterations induced by deletion on ORP8 in bone marrow cells. Indeed, ORP8 mRNA expression in liver samples of ORP8 KO BM recipients was decreased. No differences in liver CD68 mRNA or Kupffer cell numbers were observed, suggesting that ORP8 deletion likely resulted in altered macrophage function but not viability (figure S1). Whether the increased VLDL cholesterol and triglycerides in our model, as opposed to the increase in HDL cholesterol described previously is a consequence of the more pro-atherogenic phenotype of the LDLr KO recipient mice requires further study.

VLDL cholesterol is a strong inducer of atherosclerosis development, which makes the decrease in plaque development after 9 weeks of WTD feeding in our model a striking finding [24]. In line with the decreased atherosclerosis, we also found reduced foam cell formation in the peritoneal cavity of ORP8 KO BM recipients. Macrophage foam cell formation is determined by a disruption of the balance of cholesterol synthesis, cholesterol influx and cholesterol efflux. Considering the observed increased levels of VLDL cholesterol, it is unlikely that the decreased foam cell formation is due to decreased influx. Moreover, the reduced foam cell formation could not be explained by enhanced cholesterol efflux as *ex vivo* cholesterol efflux experiments failed to show significant differences. Notably ABCA1 mRNA expression was reduced both *ex vivo* in peritoneal macrophages and *in vitro* in bone marrow-derived macrophages.

Previous publications have reported an increase in ABCA1 expression when expression of ORP8 was ablated, both in THP-1 cells and in female mouse liver samples [11], [13]. In THP-1 cells, the increase in ABCA1 expression after siRNA mediated silencing of ORP8 was accompanied with increased cholesterol efflux to apoA-I [11]. In livers of female ORP8 KO mice expression of ABCA1 was also strongly increased, but this did not translate into a difference in protein expression. In contrast, in male ORP8 KO mice hepatic expression of ABCA1 showed a trend towards a decrease in expression on both RNA and on protein levels. In line, in our study the bone marrow donors were male and we observed a decrease in ABCA1 expression both in peritoneal leukocytes and in BMDMs. Lentiviral silencing of ORP8 in RAW 264.7 cells results in a modest increase in SREBP1 and SREBP2 expression, and ORP8 silencing in HuH7 cells leads to increased expression of SREBP target genes, including HMG-CoA reductase and LDLr [23], [25]. ORP8 likely affects ABCA1 expression by interfering with LXR signaling and SREBP activity [21], [23], [24]. The reduction in ABCA1 expression did not translate into differences in macrophage cholesterol efflux. ABCA1 mRNA expression is not always an accurate reflection for ABCA1 protein abundance, and previous studies on ORP8 KO liver have demonstrated changes in ABCA1 mRNA expression without corresponding changes in protein levels [13], [21]. Alternatively, the level of ABCA1 expression was still sufficient for maintaining a proper cholesterol efflux capacity under the studied conditions. In the current study the reduction in ABCA1 expression could also simply be a result of the observed reduction in lipid loading in the peritoneal cells. Previous studies in male ORP8 KO mice showed decreased expression of HMG-CoA reductase [13]. The down-regulation of HMG-CoA reductase, which is the rate limiting enzyme for cholesterol production, could result in reduced cholesterol loading. This might explain that ORP8 deletion resulted in reduced *in vivo* macrophage foam cell formation. Importantly, the reduced foam cell formation upon deletion of ORP8 in bone marrow-derived cells could have contributed to the observed reduction in lesion development. This however does not explain why macrophage ORP8 deletion primarily affects the progression of early atherosclerotic lesions, rich in macrophage foam cells, to more advance atherosclerotic lesions. *In vitro* studies using bone marrow-derived macrophages also point towards a role for ORP8 in regulation of macrophage differentiation toward different subtypes.

A study by Beaslas et al. has demonstrated that silencing of ORP8 expression in the murine RAW 264.7 macrophage cell line results in down regulation of gene pathways associated with lysosomal function, growth-factor activity and immunity Major Histocompatibility Complex type I (MCH-I) function and increased macrophage migration [25]. This could result in a reduced capacity to present antigens to immune cells such as T-cells [26]. In line, the observed decrease in CD40 and CD80 expression in ORP8 KO BMDMs indicates a reduced co-stimulatory potential and a decreased capacity to activate T-cells [26]. In the current study we also demonstrated a decrease in mRNA expression of IL-6 and excretion of TNFα and IL-6 by LPS-stimulated M1 BMDMs lacking ORP8. Furthermore, mRNA expression of the M2 markers YM-1 and FIZZ-1 was decreased. Upon deletion of ORP8, macrophages thus show a reduced polarization towards both M1 and M2 subtypes [27]. The effects of ORP8 deficiency on macrophage inflammatory signaling could provide a mechanistic explanation for the increase in VLDL levels in mice lacking macrophage ORP8. However, decreased excretion of TNFα and IL-6 would lead to decreased synthesis of cholesterol, triglycerides and VLDL particles [28]. Therefore it is unlikely that the observed increase in VLDL levels in ORP8 KO BM recipients can be attributed to a decrease in production of pro-inflammatory cytokines by Kupffer cells lacking ORP8.

Importantly, macrophage differentiation plays a crucial role in the advanced stages of atherosclerotic plaque development. “Wound healing” M2 macrophages promote plaque stability, whereas “classically activated” M1 macrophages contribute to the thinning of the fibrous cap [29]. Both types of macrophages can be found in advanced plaques, creating a complex dynamic equilibrium that determines the structure of the plaque and its likelihood to rupture [18], [29], [30], [31]. In human plaques, expression of ORP8 is localized in the shoulder region [11]. Research on human carotid artery sections has shown that macrophages in the shoulder region of the plaque strongly exhibit markers of M1 macrophages such as iNOS and HLA [29]. Moreover, an increase in TNFα and IL-6 mRNA is observed in ruptured plaque segments [29]. By producing pro-inflammatory factors such as TNFα and IL-6, M1 macrophages are able to drive the inflammatory reaction and thereby enhance the development of atherosclerosis [32], [33], [34], [35]. The observed decrease in the production of TNFα and IL-6 and reduced expression of co-stimulatory molecules by macrophages lacking ORP8 is thus expected to lead to reduced inflammatory activity in the plaque.

In summary, ORP8 KO macrophages have a decreased ability to drive atherosclerosis due to lowered production of pro-inflammatory cytokines and a reduced formation of foam cells. In concert, these two effects may result in a reduced size of atherosclerotic plaques in ORP8 KO BM recipients after 9 weeks of WTD feeding, despite the observed increase in VLDL cholesterol and triglycerides.

## Supporting Information

Figure S1 **Transplantation of LDLr KO mice with ORP8 KO bone marrow (BM) results in decreased expression of ORP8 in the liver, but no changes in Kupffer cell content.** Livers were isolated at 17 weeks after transplantation and after 9 weeks of WTD feeding. A) A reduction in RNA expression of ORP8 as a fraction of housekeeping (HK) in liver lysates was measured by QPCR (* P<0.05). B) No difference in expression of the macrophage marker CD68 could be detected using QPCR. C) Liver sections were stained for F4/80 and F4/80 positive cells were quantified as percentage of total cells counted. No difference in liver F4/80 positive cells could be found. D) Representative images of F4/80 stained liver sections. Original magnification 40×. Significance was determined by Student T-test.
(TIF)
