# Supplementary figures and images for: Orp8 Deficiency in Bone Marrow-Derived Cells Reduces Atherosclerotic Lesion Progression in LDL Receptor Knockout Mice
Source: PLoS One. 2014 Oct 27;9(10):e109024. doi: 10.1371/journal.pone.0109024 (PMC4209969; doi:10.1371/journal.pone.0109024)

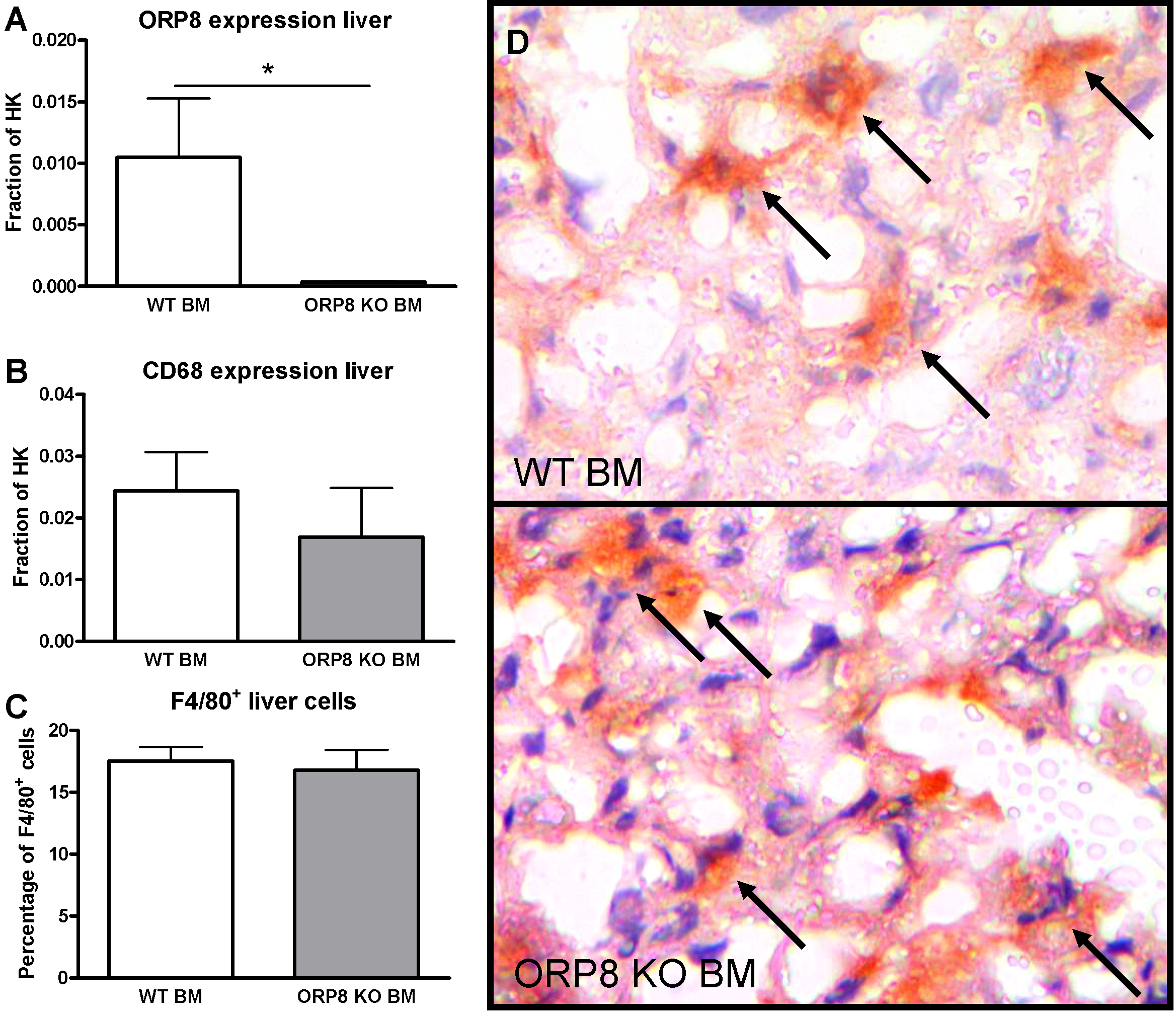

Supplement: Figure S1 — Transplantation of LDLr KO mice with ORP8 KO bone marrow (BM) results in decreased expression of ORP8 in the liver, but no changes in Kupffer cell content. Livers were isolated at 17 weeks after transplantation and after 9 weeks of WTD feeding. A) A reduction in RNA expression of ORP8 as a fraction of housekeeping (HK) in liver lysates was measured by QPCR (* P<0.05). B) No difference in expression of the macrophage marker CD68 could be detected using QPCR. C) Liver sections were stained for F4/80 and F4/80 positive cells were quantified as percentage of total cells counted. No difference in liver F4/80 positive cells could be found. D) Representative images of F4/80 stained liver sections. Original magnification 40×. Significance was determined by Student T-test. (TIF) [file pone.0109024.s001.tif]
